# Gender and contemporary risk of adverse events in atrial fibrillation

**DOI:** 10.1093/eurheartj/ehae539

**Published:** 2024-09-01

**Authors:** Asgher Champsi, Alastair R Mobley, Anuradhaa Subramanian, Krishnarajah Nirantharakumar, Xiaoxia Wang, David Shukla, Karina V Bunting, Inge Molgaard, Jeremy Dwight, Ruben Casado Arroyo, Harry J G M Crijns, Luigina Guasti, Maddalena Lettino, R Thomas Lumbers, Bart Maesen, Michiel Rienstra, Emma Svennberg, Otilia Țica, Vassil Traykov, Stylianos Tzeis, Isabelle van Gelder, Dipak Kotecha

**Affiliations:** Institute of Cardiovascular Sciences, University of Birmingham, Medical School, Vincent Drive, Birmingham B15 2TT, UK; West Midlands NHS Secure Data Environment, University Hospitals Birmingham NHS Foundation Trust, Mindelsohn Way, Birmingham B15 2TH, UK; NIHR Birmingham Biomedical Research Centre, Institute of Translational Medicine, Queen Elizabeth Hospital, Heritage Building, Mindelsohn Way, Birmingham B15 2TH, UK; Institute of Cardiovascular Sciences, University of Birmingham, Medical School, Vincent Drive, Birmingham B15 2TT, UK; West Midlands NHS Secure Data Environment, University Hospitals Birmingham NHS Foundation Trust, Mindelsohn Way, Birmingham B15 2TH, UK; NIHR Birmingham Biomedical Research Centre, Institute of Translational Medicine, Queen Elizabeth Hospital, Heritage Building, Mindelsohn Way, Birmingham B15 2TH, UK; Institute of Applied Health Research, University of Birmingham, University Road West, Birmingham B15 2TT, UK; West Midlands NHS Secure Data Environment, University Hospitals Birmingham NHS Foundation Trust, Mindelsohn Way, Birmingham B15 2TH, UK; NIHR Birmingham Biomedical Research Centre, Institute of Translational Medicine, Queen Elizabeth Hospital, Heritage Building, Mindelsohn Way, Birmingham B15 2TH, UK; Institute of Applied Health Research, University of Birmingham, University Road West, Birmingham B15 2TT, UK; Institute of Cardiovascular Sciences, University of Birmingham, Medical School, Vincent Drive, Birmingham B15 2TT, UK; NIHR Birmingham Biomedical Research Centre, Institute of Translational Medicine, Queen Elizabeth Hospital, Heritage Building, Mindelsohn Way, Birmingham B15 2TH, UK; West Midlands NHS Secure Data Environment, University Hospitals Birmingham NHS Foundation Trust, Mindelsohn Way, Birmingham B15 2TH, UK; Institute of Applied Health Research, University of Birmingham, University Road West, Birmingham B15 2TT, UK; Institute of Cardiovascular Sciences, University of Birmingham, Medical School, Vincent Drive, Birmingham B15 2TT, UK; NIHR Birmingham Biomedical Research Centre, Institute of Translational Medicine, Queen Elizabeth Hospital, Heritage Building, Mindelsohn Way, Birmingham B15 2TH, UK; Patient & Public Representatives, European Society of Cardiology, Sophia Antipolis, France; Patient & Public Representatives, European Society of Cardiology, Sophia Antipolis, France; Department of Cardiology, H.U.B.-Hôpital Erasme, Université Libre de Bruxelles, Brussels 1070, Belgium; Department of Cardiology, Maastricht University Medical Center, Maastricht, The Netherlands; Cardiovascular Research Institute (CARIM), Maastricht University, Maastricht, The Netherlands; Department of Medicine and Surgery, University of Insubria, Varese, Italy; Cardiothoracic and Vascular Department, Fondazione IRCCS San Gerardo dei Tintori, Monza, Italy; Institute of Health Informatics, University College London, London, UK; NIHR University College London Hospitals Biomedical Research Centre, London, UK; Department of Cardiology, Maastricht University Medical Center, Maastricht, The Netherlands; Cardiovascular Research Institute (CARIM), Maastricht University, Maastricht, The Netherlands; Department of Cardiology, University of Groningen, University Medical Centre Groningen, Groningen, The Netherlands; Department of Medicine Huddinge, Karolinska Institutet, Karolinska University Hospital, Stockholm, Sweden; Institute of Cardiovascular Sciences, University of Birmingham, Medical School, Vincent Drive, Birmingham B15 2TT, UK; Cardiology Department, Emergency County Clinical Hospital of Oradea, Oradea, Romania; Clinic of Cardiology, Acibadem City Clinic Tokuda University Hospital, Sofia, Bulgaria; Cardiology Department, Mitera Hospital, Athens, Greece; Department of Cardiology, University of Groningen, University Medical Centre Groningen, Groningen, The Netherlands; Institute of Cardiovascular Sciences, University of Birmingham, Medical School, Vincent Drive, Birmingham B15 2TT, UK; West Midlands NHS Secure Data Environment, University Hospitals Birmingham NHS Foundation Trust, Mindelsohn Way, Birmingham B15 2TH, UK; NIHR Birmingham Biomedical Research Centre, Institute of Translational Medicine, Queen Elizabeth Hospital, Heritage Building, Mindelsohn Way, Birmingham B15 2TH, UK

**Keywords:** Atrial fibrillation, Gender, Women, Sex, Stroke, Thromboembolism

## Abstract

**Background and Aims:**

The role of gender in decision-making for oral anticoagulation in patients with atrial fibrillation (AF) remains controversial.

**Methods:**

The population cohort study used electronic healthcare records of 16 587 749 patients from UK primary care (2005–2020). Primary (composite of all-cause mortality, ischaemic stroke, or arterial thromboembolism) and secondary outcomes were analysed using Cox hazard ratios (HR), adjusted for age, socioeconomic status, and comorbidities.

**Results:**

78 852 patients were included with AF, aged 40–75 years, no prior stroke, and no prescription of oral anticoagulants. 28 590 (36.3%) were women, and 50 262 (63.7%) men. Median age was 65.7 years (interquartile range 58.5–70.9), with women being older and having other differences in comorbidities. During a total follow-up of 431 086 patient-years, women had a lower adjusted primary outcome rate with HR 0.89 vs. men (95% confidence interval [CI] 0.87–0.92; *P* < .001) and HR 0.87 after censoring for oral anticoagulation (95% CI 0.83–0.91; *P* < .001). This was driven by lower mortality in women (HR 0.86, 95% CI 0.83–0.89; *P* < .001). No difference was identified between women and men for the secondary outcomes of ischaemic stroke or arterial thromboembolism (adjusted HR 1.00, 95% CI 0.94–1.07; *P* = .87), any stroke or any thromboembolism (adjusted HR 1.02, 95% CI 0.96–1.07; *P* = .58), and incident vascular dementia (adjusted HR 1.13, 95% CI 0.97–1.32; *P* = .11). Clinical risk scores were only modest predictors of outcomes, with CHA_2_DS_2_-VA (ignoring gender) superior to CHA_2_DS_2_-VASc for primary outcomes in this population (receiver operating characteristic curve area 0.651 vs. 0.639; *P* < .001) and no interaction with gender (*P* = .45).

**Conclusions:**

Removal of gender from clinical risk scoring could simplify the approach to which patients with AF should be offered oral anticoagulation.

## Structured Graphical Abstract

**Structured Graphical Abstract:**
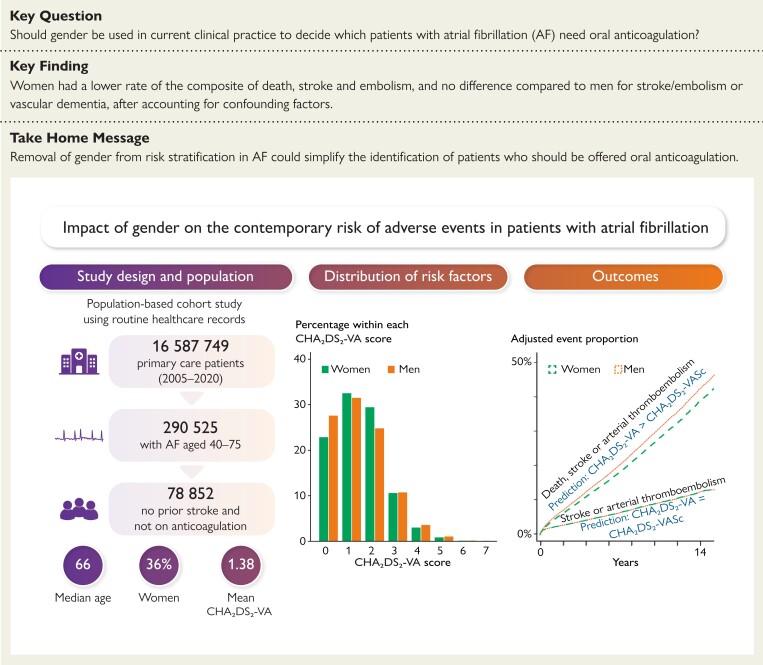
The impact of gender on adverse events in patients with atrial fibrillation (AF) based on a population cohort study using electronic healthcare records from UK primary care (2005–20).

**See the editorial comment for this article ‘CHA_2_DS_2_-VASc or a non-sex score (CHA_2_DS_2_-VA) for stroke risk prediction in atrial fibrillation: contemporary insights and clinical implications', by G.Y.H. Lip *et al*., https://doi.org/10.1093/eurheartj/ehae540.**

## Introduction

Atrial fibrillation (AF) remains a common, costly, and high-morbidity condition impacting patients across the whole spectrum of healthcare. The high and ultimately preventable risk of stroke and other thromboembolic events associated with AF^1^ has driven the generation of clinical risk scores to help determine which patients would benefit from oral anticoagulation. These range from simple clinical classification systems, which have dominated routine practice,^2,3^ to more complex algorithms^4^ and the use of biomarkers.^5^ However, most clinical risk scores have broadly similar performance and may not accurately predict those that will go on to suffer from strokes, may not account for the use of direct oral anticoagulants (DOACs), and ignore other thromboembolic outcomes, such as vascular dementia.

**Figure 1:**
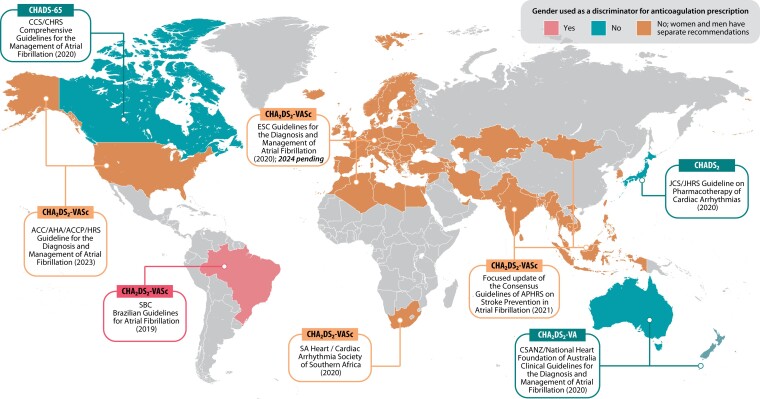
Variation in global use of gender for risk stratification in atrial fibrillation. Guidelines from different global regions showing marked variability in the use of gender as a discriminating factor for the prescription of oral anticoagulation in patients with AF. Further details are presented in Supplementary data online, *Table S1*. CCS = Canadian Cardiovascular Society; CHRS = Canadian Heart Rhythm Society; ESC = European Society of Cardiology; AHA = American Heart Association; ACC = American College of Cardiology; ACCP = American College of Clinical Pharmacy; HRS = Heart Rhythm Society; JCS = Japanese Circulation Society; JHRS = Japanese Heart Rhythm Society; SBC = Brazilian Society of Cardiology; SA Heart = South African Heart Association; APHRS = Asia-Pacific Heart Rhythm Society; CSANZ = Cardiac Society of Australia and New Zealand

A further challenge with AF risk scores has been their inclusion of gender as a risk stratifier. Higher rates of stroke in women with AF have been reported in historical data,^6^ although this is likely confounded by the contribution of other risk factors. This includes older age and lower anticoagulation rates in women and higher mortality in men, which is a competing risk for stroke. More recently, gender has been reconsidered as a risk modifier;^7,8^ however, international guidelines vary considerably (*Figure 1*; Supplementary data online, *Table S1*). The inclusion of gender in risk scores has typically been circumvented by using different risk cut-offs for each gender, for example, a CHA_2_DS_2_-VASc score of 2 for men, but 3 for women, to qualify for a class I indication for oral anticoagulation.

This population cohort study was performed to address a key evidence gap in patients with AF, where gender plays a role in the decision for anticoagulation. The study specifically excluded those with prior stroke or age ≥75 years where there is strong confounding of clinical outcomes and guideline-recommended indication for oral anticoagulation, irrespective of the patient's gender. The aim of this study was to provide real-world evidence on the value of gender for risk stratification in contemporary patients with AF where anticoagulation is being considered.

## Methods

### Study design and data source

A population-based, retrospective cohort study was conducted between 1 January 2005 and 31 December 2020 using data obtained from the IQVIA Medical Research Database (IMRD), a proprietary database of Cegedim SA (France). IMRD is a primary care database containing pseudonymized medical records of patients registered within general practices across the UK using the VISION clinical system.^9^ IMRD comprises over 18 million patient records from 832 general practices in the UK, representing a snapshot of around 6% of the UK population. The database contains information on patient demographics and coded records of diagnoses using the Read code clinical classification system, dispensed prescriptions, and additional health information, such as physical and biochemical measurements. The primary care coded database is used for billing and reimbursement purposes in the UK National Health Service (NHS), with high data quality incentivized through the Quality and Outcomes Framework.^10^ Data extraction was conducted using the DExtER software.^11^ This study meets all five of the CODE-EHR framework minimum standards for the use of structured healthcare data in clinical research, with three out of five standards meeting the preferred criteria; see Supplementary data online, *Table S2* for CODE-EHR domains and Appendix 1 for the CODE-EHR checklist.^12^ All codes used in this study were predefined and pre-published for transparency and re-use by other researchers in concordance with the CODE-EHR framework.

### Ethics

Data collection for IMRD was approved by the NHS South-East Multicentre Research Ethics Committee in 2003. Under the terms of this approval, each study protocol undergoes independent review from the Scientific Review Committee, with approval obtained in July 2017 (SRC reference number: SRC 17THIN061).

### Study population

Practices were considered eligible 1 year after the establishment of the VISION clinical system within their practice or 1 year after reporting mortality rates comparable to national averages,^13^ whichever was the latest. In eligible general practices, adults aged 40 years or older and registered during the study period for at least a year were included. For patients with an existing AF diagnosis, the index date was assigned as the date of patient eligibility (1 year after their registration date with an eligible general practice). For patients with a new diagnosis of AF after they became eligible, the index date was assigned as the date of AF diagnosis.

### Exclusions

Individuals aged ≥75 years or with a history of stroke in their medical record were excluded as these patients have an undisputed indication for oral anticoagulation for stroke prevention reasons regardless of gender. In addition, patients with an active prescription for a vitamin K antagonist or DOAC were excluded irrespective of stroke risk assessment.

### Covariates

This study uses the term gender as it relates to personal identity and recording of such within the patient's medical record. Gender is documented as female or male, with no current option to record transgender status or specify sex at birth. Age, socioeconomic status, and diagnoses of hypertension, diabetes mellitus, heart failure, and vascular disease were considered as confounders. Age was modelled as a continuous variable. Socioeconomic deprivation was recorded as the Townsend deprivation index categorized into quintiles,^14^ with missing data specifically encoded as such to avoid embedding bias. The listed comorbidities were extracted from the medical record according to the pre-published coding scheme. The CHA_2_DS_2_-VASc score was calculated with 2 points given for prior stroke, transient ischaemic attack or thromboembolism, 2 points for age ≥75 years, and 1 point for heart failure, hypertension, age 65–74 years, diabetes mellitus, vascular disease, or female gender. The CHA_2_DS_2_-VA score was similarly calculated, but without considering gender.

### Follow-up and outcomes

The primary outcome was the composite of all-cause mortality, ischaemic stroke, or arterial thromboembolism. Including mortality within the primary outcome was essential as death is a competing risk for thromboembolic events (dead patients cannot be admitted with a stroke), and mortality risks are higher in men, leading to further bias. Secondary outcomes were: (1) ischaemic stroke or arterial thromboembolism; (2) any stroke (ischaemic or haemorrhagic) or any thromboembolism (arterial or venous); (3) vascular dementia; and (4) all-cause mortality. Strokes with an unspecified cause were included in the ischaemic category. Outcomes were considered from the index date until the earliest of the following time points: (1) recording of the outcome of interest; (2) patient censorship due to death or de-registration from their registered practice; (3) practice censorship due to ceasing of their data contribution to IMRD; and (4) study end date of 31 December 2020.

### Statistical analysis

Summary results are presented as percentages, median, and interquartile range (IQR; displayed as 25th to 75th quartiles), or mean and standard deviation (SD). Group comparisons were made using the Kruskal–Wallis non-parametric rank test adjusted for multiple comparisons. Outcomes were analysed using Cox proportional hazards regression models for women vs. men reported for univariate analysis and multivariate adjustment for the aforementioned confounders. Hazard ratios (HR) and 95% confidence intervals (CI) are presented, along with corresponding *P*-values. Proportional hazards were tested using a log–log plot of scaled Schoenfeld residuals to ensure that the hazard related to gender remained constant over time. Effect modification was assessed using *P*-values from interaction terms fitted in the multivariable models. Kaplan–Meier plots were used to graph the unadjusted outcomes according to gender, and failure plots to present the adjusted data from the multivariate model. The interaction of age as a continuous variable with gender on the primary outcome was assessed using cubic splines in the Cox model and a Royston–Parmar flexible parametric survival model.^15^ Two pre-defined sensitivity analysis were conducted for the primary outcome: (1) censoring at the time of treatment with any oral anticoagulant; and (2) censoring for patients with incident AF only. Post-hoc analyses were: (1) competing risk for ischaemic stroke or arterial thromboembolism with death using the method of Fine and Gray; and (2) separation into three time periods (index AF date 2005–09, 2010–14 and 2015–20) for assessment of the primary outcome with censoring at 1 year.

The area under the receiver operating characteristic curve (AUROC) was determined using logistic regression, with group comparisons using a *χ*^2^ test. Robust methods for model comparisons are presented in the online supplement. Net reclassification improvement and integrated discrimination improvement were evaluated to assess the impact of gender on risk prediction for the primary outcome; bootstrapping to calculate CI was not required due to the lack of any reclassification.

A two-tailed *P*-value of .05 was considered statistically significant. Analyses were performed on Stata Version 17 (StataCorp LP, College Station, TX, USA).

## Results

A total of 16 587 749 patients from 828 eligible primary care practices in the UK were evaluated, of which 5 199 994 were eligible and aged 40–75 years, including 290 525 with an AF diagnosis code (5.6%). In total, 78 852 patients had AF, were aged 40–75 years, had no prior stroke, and were not prescribed oral anticoagulants (see Supplementary data online, *Figure S1*). There were 28 590 women (36.3%) and 50 262 men (63.7%). Median age was 65.7 years (IQR 58.5–70.9), with women older by a median difference of 2.5 years compared to men. Women had higher rates of coexisting hypertension and lower rates of heart failure, diabetes, and vascular disease compared to men (*Table 1*). All comparisons between women and men were statistically significant (*P* < .001). The mean CHA_2_DS_2_-VASc and CHA_2_DS_2_-VA scores were 1.74 (SD 1.27) and 1.38 (SD 1.16), respectively. There was a statistical, but not a clinically significant difference in CHA_2_DS_2_-VA scores between women and men (mean 1.42 vs. 1.36; *P* < .0001), and the distribution across scores was similar (*Structured Graphical Abstract*). The total follow-up period for outcome assessment was 159 355 person-years for women (mean 5.6 years per-patient; SD 4.1) and 271 731 person-years for men (mean 5.4 years per-patient; SD 4.0).

**Table 1:** Baseline demographics by gender

Baseline characteristic	All (*n* = 78 852)	Women (*n* = 28 590)	Men (*n* = 50 262)
Age, median years (IQR)	65.7 (58.5–70.9)	67.3 (60.6–71.6)	64.8 (57.4–70.4)
Women, *n* (%)	28 590 (36.3%)		
CHA_2_DS_2_-VASc, mean score (SD)	1.74 (1.27)	2.42 (1.12)	1.36 (1.19)
CHA_2_DS_2_-VA, mean score (SD)	1.38 (1.16)	1.42 (1.12)	1.36 (1.19)
Hypertension, *n* (%)	36 478 (46.3%)	14 058 (49.2%)	22 420 (44.6%)
Heart failure, *n* (%)	5704 (7.2%)	1698 (5.9%)	4006 (8.0%)
Diabetes mellitus, *n* (%)	11 411 (14.5%)	3763 (13.2%)	7648 (15.2%)
Vascular disease, *n* (%)	8275 (10.5%)	2034 (7.1%)	6241 (12.4%)
Townsend deprivation score, *n* (%)			
Quintile 1 (least deprived)	17 692 (22.4%)	6151 (21.5%)	11 541 (23.0%)
Quintile 2	16 102 (20.4%)	5721 (20.0%)	10 381 (20.7%)
Quintile 3	14 421 (18.3%)	5288 (18.5%)	9133 (18.2%)
Quintile 4	11 706 (14.9%)	4480 (15.7%)	7226 (14.4%)
Quintile 5 (most deprived)	8086 (10.3%)	3094 (10.8%)	4992 (9.9%)
Missing deprivation data	10 845 (13.8%)	3856 (13.5%)	6989 (13.9%)

### Primary outcome

**Figure 2:**
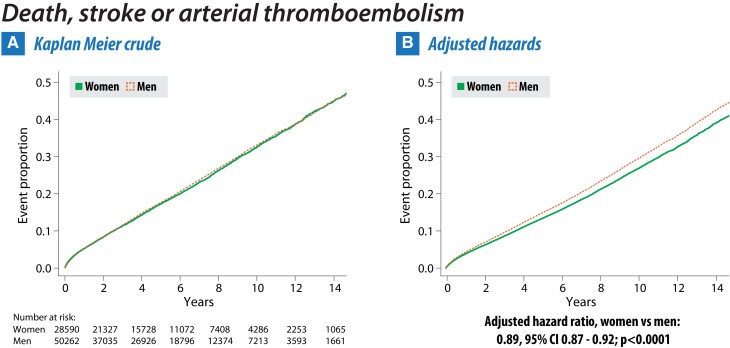
Crude and adjusted primary outcome by gender. Cumulative event curves for the composite of all-cause mortality, ischaemic stroke, or arterial thromboembolism for women (solid green line) and men (dashed orange line). Presented as crude Kaplan–Meier curves (panel A) and after adjustment for age, socioeconomic deprivation status, and diagnoses of hypertension, diabetes mellitus, heart failure, and vascular disease (panel B)

The composite of all-cause mortality, ischaemic stroke, or arterial thromboembolism occurred in 6172 women (21.6%) and 10 721 men (21.3%). There was no difference between women and men in univariate analysis (HR .98, 95% CI .96–1.02; *P* = .37; *Table 2*), with superimposed Kaplan–Meier curves (*Figure 2*). After adjustment for age, socioeconomic status, and comorbidities, women had a lower rate of the primary outcome, with adjusted HR .89 vs. men (95% CI .87–.92; *P* < .001).

**Table 2:** Primary and secondary outcomes

Outcome	Events, *n* (%)		Unadjusted event rate, per 1000 person-years		Women vs. men	
	Women (*n* = 28 590)	Men (*n* = 50 262)	Women (159 355 person-years of follow-up)	Men (271 731 person-years of follow-up)	Unadjusted hazard ratio (95% CI)	Adjusted hazard ratio^a^ (95% CI)
All-cause mortality, ischaemic stroke, or arterial thromboembolism	6172 (21.6%)	10 721 (21.3%)	38.7	39.5	.98 (.96–1.02); *P* = .37	.89 (.87–.92); *P* < .001
Ischaemic stroke or arterial thromboembolism	1467 (5.1%)	2288 (4.6%)	9.2	8.4	1.10 (1.03–1.17); *P* = .004	1.00 (.94–1.07); *P* = .87
Any stroke (ischaemic or haemorrhagic) or any thromboembolism (arterial or venous)	2186 (7.7%)	3417 (6.8%)	13.7	12.6	1.10 (1.04–1.16); *P* < .001	1.02 (.96–1.07); *P* = .58
Vascular dementia	323 (1.1%)	380 (0.8%)	2.0	1.4	1.44 (1.24–1.67); *P* < .001	1.13 (.97–1.32); *P* = .11
All-cause mortality	5079 (17.8%)	9090 (18.1%)	31.9	33.4	.95 (.92–.99); *P* = .005	.86 (.83–.89); *P* < .001

^a^Adjusted for age, socioeconomic deprivation status, and diagnoses of hypertension, diabetes mellitus, heart failure, and vascular disease.

During follow-up, 17 133 women (60.0%) and 30 307 men (60.3%) received oral anticoagulants (*P* = .31 for comparison). In a sensitivity analysis that censored patients at the time of commencement of oral anticoagulation, there was no impact on results for the primary outcome with adjusted HR of .87 for women vs. men (95% CI .83–.91; *P* < .001; Supplementary data online, *Figure S2*). Results for those with incident AF (*n* = 57 107) were the same as the total population of any AF exposure, with unadjusted HR .98 for women vs. men (95% CI .95–1.02; *P* = .37) and adjusted HR .92 (95% CI .89–.96; *P* < .001). A post-hoc analysis demonstrated similar 1-year event rates after adjusting for risk factors when comparing 2005–09, 2010–14, and 2015–20 (see Supplementary data online, *Figure S3*).

### Secondary outcomes

**Figure 3:**
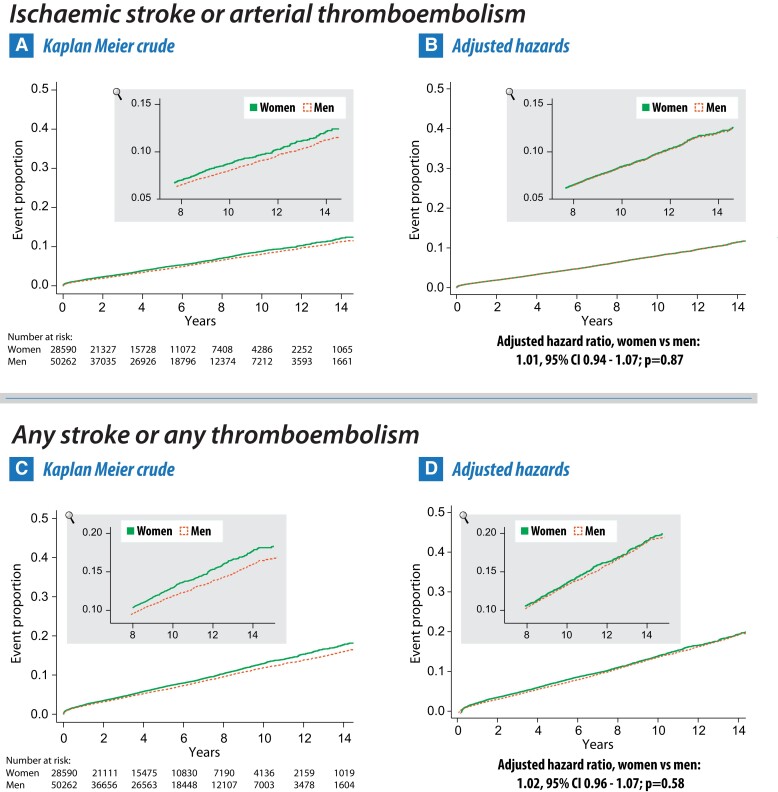
Crude and adjusted secondary outcomes by gender. Cumulative event curves for ischaemic stroke or arterial thromboembolism and any stroke (ischaemia or haemorrhagic) or any thromboembolism (arterial or venous). Presented as crude Kaplan–Meier curves (panels A and C) and after multivariate adjustment (panels B and D) for women (solid green line) and men (dashed orange line)

There were numerically more events in women for ischaemic stroke or arterial thromboembolism, and any stroke or any thromboembolism, with a 10% increased hazard in women for both outcomes compared with men in crude analysis (*Table 2* and *Figure 3*). After adjusting for confounders, no difference was identified between women and men for either outcome (HR 1.00, 95% CI .94–1.07, *P* = .87 and 1.02, 95% CI .96–1.07, *P* = .58). The lack of difference between genders was confirmed in a post-hoc analysis to account for competing risk between ischaemic stroke or arterial thromboembolism and death (HR 1.03, 95% CI .96–1.10; *P* = .40).

Vascular dementia followed the same pattern as thromboembolic outcomes, with no significant difference between women and men after risk factor adjustment (HR 1.13, 95% CI .97–1.32; *P* = .11; *Table 2* and Supplementary data online, *Figure S4*).

Death occurred in 14 169 patients (18.0%) during follow-up, with a rate of 31.9 per 1000 patient-years in women and 33.5 per 1000 patient-years in men. All-cause mortality rates were significantly lower in women after adjusting for age, socioeconomic status, and comorbidities (HR .86, 95% CI .83–.89; *P* < .001) (*Table 2* and Supplementary data online, *Figure S4*).

### Comparison of risk scoring with and without gender

**Figure 4:**
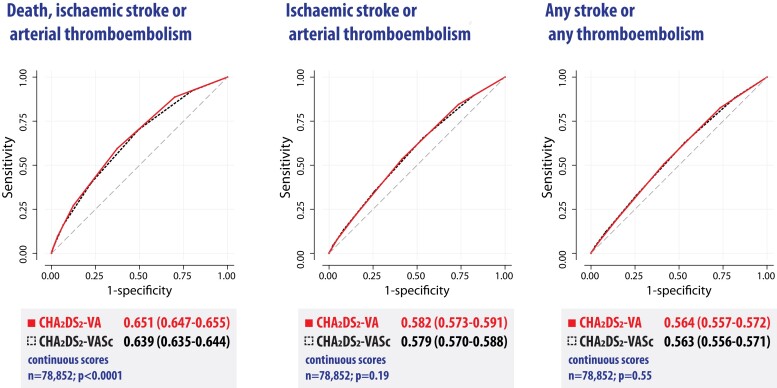
Comparison of risk scores with and without gender. Comparison of the area under the receiver operator characteristic curve for the CHA_2_DS_2_-VA score (solid red line) and CHA_2_DS_2_-VASc score (dashed black line) for each outcome. Higher values indicate better accuracy, with the dashed grey line indicating accuracy no better than chance. Note patients with prior stroke or age ≥75 years were excluded to focus on a population where gender was a contributor to decision-making on oral anticoagulation; hence, these performance figures do not reflect the standard use of these risk scores

CHA_2_DS_2_-VA and CHA_2_DS_2_-VASc were only modest predictors of adverse outcomes in this selected cohort of patients with AF, with AUROC values consistently showing relatively poor discrimination. As a continuous score, CHA_2_DS_2_-VA was superior to CHA_2_DS_2_-VASc for the primary outcome with AUROC .651 vs. .639 (*P* < .001) (*Figure 4*). Further robust comparison is presented in the online supplement. CHA_2_DS_2_-VA was also superior to CHA_2_DS_2_-VASc when used as a categorical score (2 or above), with AUROC .611 vs. .604 (*P* < .001) (see Supplementary data online, *Figure S5*). There were no differences between CHA_2_DS_2_-VA and CHA_2_DS_2_-VASc for ischaemic stroke or arterial thromboembolism, and any stroke or any thromboembolism.

The CHA_2_DS_2_-VA score as a continuous variable was superior to age alone using a cut-off of 65 years, with AUROC .651 vs. .618 (*P* < .001). This was not the case when using CHA_2_DS_2_-VA as a categorical score (2 or above), with AUROC .611 vs. .618 for age 65 years (*P* = .009) (see Supplementary data online, *Figure S6*).

**Figure 5:**
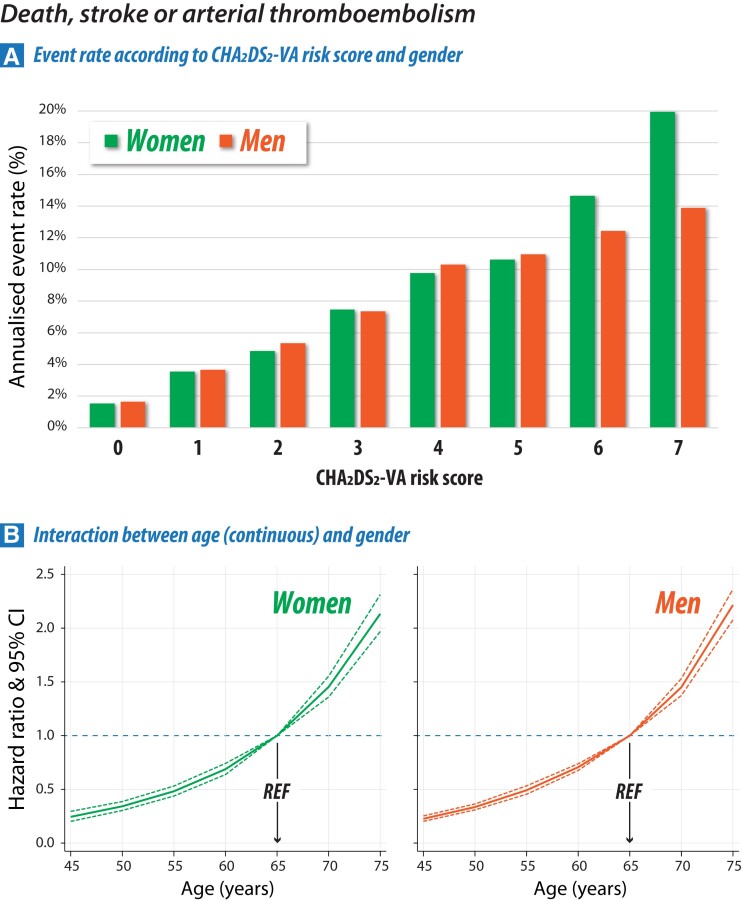
Primary outcome according to risk stratification. (A) Annualised crude event rate for the composite of all-cause mortality, ischaemic stroke, or arterial thromboembolism for each CHA_2_DS_2_-VA score according to gender; refer to Supplementary data online, *Figure S7* for the secondary outcome of ischaemic stroke or arterial thromboembolism. (B) Age as a continuous variable using a cubic spline model in reference to age = 65 years and presented separately for women and men

Other components of clinical risk scoring (heart failure, hypertension, diabetes, and vascular disease) were individually associated with higher risk of the primary outcome (see Supplementary data online, *Figure S7*). For each 1 point increase in CHA_2_DS_2_-VA score, the hazard of all-cause mortality, ischaemic stroke, or arterial thromboembolism increased by 1.48 (95% CI 1.46–1.50; *P* < .001; Supplementary data online, *Figure S8*). There was no interaction noted between CHA_2_DS_2_-VA as a continuous score and gender (*P* = .45). Except for those at the highest risk, crude primary outcome event rates were similar between women and men in each CHA_2_DS_2_-VA score categories, with an annualized rate of 3.56% and 3.66% for CHA_2_DS_2_-VA = 1 and 4.84% and 5.33% for CHA_2_DS_2_-VA = 2 (*Figure 5A*; Supplementary data online, *Figure S9* for ischaemic stroke/arterial thromboembolism). No reclassification was seen with the addition of gender to CHA_2_DS_2_-VA for either cases (death, ischaemic stroke, or arterial thromboembolism) or controls (no primary outcome events). Net reclassification improvement was zero when gender was added to the model, and integrated discrimination improvement was not significant (*P* > .5). There was no difference between women and men in the association of age (as a continuous variable) with primary outcome events (*Figure 5B*).

## Discussion

This study used a large, contemporary, primary care population to assess the impact of gender on adverse outcomes in patients with AF. To approximate the population where gender could potentially play a role in treatment selection for oral anticoagulation, those with a prior stroke or age ≥75 years were specifically excluded, as there is already a clear indication in these groups for oral anticoagulation regardless of gender. After accounting for various confounders, including age, comorbidities, anticoagulation use, and the differential rate of death, there was no indication in this study that gender should play a major part in risk stratification for anticoagulation therapy. There was no difference between women and men in this population for different types of stroke or different types of thromboembolism, with higher age in women likely offsetting the greater vascular comorbidity burden in men. Mortality rates and, hence, the incidence of the composite primary outcome were overall higher in men than women. CHA_2_DS_2_-VA (ignoring gender) had better performance than CHA_2_DS_2_-VASc in this selected population, although both scores can oversimplify treatment decisions and have limited accuracy for prediction of adverse outcomes in individual patients.

Gender has always been a controversial issue with regard to decisions on prevention of stroke and thromboembolism in the context of AF. It became part of routine practice in 2010 after validation of the CHA_2_DS_2_-VASc score in 1084 patients from the 2003–04 Euro Heart Survey of hospitalized patients with AF.^3^ The association between female gender and ischaemic stroke is changing over time, with a large registry cohort finding that the incidence of ischaemic stroke in more recent years was no longer different between women and men.^16^ Numerous cohort studies have validated the CHA_2_DS_2_-VASc score in different populations and against other risk scores;^17–19^ however, the issue of gender has never been settled. International guideline committees have tended to get around the issue by suggesting different cut-off points for women and men (*Figure 1*).^20,21^ It is possible that this may have inadvertently contributed in the past to lower reported rates of appropriate anticoagulation in women.^22,23^ Of note, using a score for risk assessment may be different from threshold-based decisions for oral anticoagulation.

The association between gender and outcomes in AF is confounded by substantial differences in age, comorbidity burden, symptoms, and access to interventional therapy when comparing women with men.^24^ In addition, comorbidities and risk factors are known to change over time. This study adjusted for relevant clinical factors that may have impacted previous observational studies,^7^ and as a result, we saw similar event rates over different time-periods. Substantive differences were noted between the unadjusted and adjusted analyses for every outcome, highlighting the dependence of prognosis on individual patient profiles and the importance of considering these confounders. The differential rate of death amongst women and men is also important to consider as dying precludes the possibility of developing a stroke or another thromboembolic event. This is of particular relevance in older multimorbid populations (such as patients with AF), and why death was included within the primary outcome of this study. In recent years, the complexities of gender identity have led to new challenges, with the potential for transgender patients to not receive appropriate therapy, even though they have high rates of cardiovascular events.^25^ Removing all aspects of gender from risk stratification in AF could have additional benefit on securing equality in the provision of evidence-based therapy.

The accuracy of risk scores and their relatively poor ability to discriminate patients who go on to suffer from the sequelae of AF is a concern. Most clinical risk scores for stroke prevention in AF have AUROC values of .6–.7, indicating that a substantial number of patients will not be appropriately classified, and the chance of missing people where oral anticoagulation could have prevented thromboembolic events. The median AUROC for CHA_2_DS_2_-VASc in a meta-analysis of eight studies was .600 as used in clinical practice (i.e. as a categorical cut-off).^26^ Of note, AUROC values of .5 indicate that the risk model is no better than a random guess or toss of a coin. Although the main objective of this study was to assess the value of gender in risk profiling, our results also confirm that stratification based on clinical categories is far from ideal. Attempts to improve these scores have led to more complex calculators^27^ and the inclusion of biomarkers to refine risk assessment.^28^ These approaches have not been as widely validated, and the transition away from simple clinical scores may have unintended consequences or lead to health inequalities. Healthcare professionals and patients should be made aware of the poor performance of available risk scores and seek to personalize prescription of oral anticoagulation where possible. This includes considering the broad range of other clinical factors that may modulate thromboembolic risk in AF and could contribute to decision-making on oral anticoagulation, such as kidney disease.^29^ Robust evidence for clinical risk scores from randomized trials is lacking, with a cluster randomized study of automated CHA_2_DS_2_-VASc to advise on anticoagulant prescription finding no difference in thromboembolic outcomes compared to usual care,^30^ and a biomarker-guided approach still under evaluation (NCT03753490). Other ongoing trials are exploring the use of DOACs in younger populations at lower established risk (DaRe2THINK, NCT04700826^31^; BRAIN-AF, NCT02387229^32^), which may in the future remove the need for risk scores entirely. Although lifetime risk of AF is similar in women and men, AF onset occurs around 10 years later in women,^33^ making the feasibility of trials uncertain to address the question of gender in low or intermediate risk patients.

Observational datasets are prone to reflect prescription biases common in routine clinical practice, and larger sample sizes do not necessarily ameliorate these effects.^34^ This contemporary study showed a lower mortality in women after careful multivariable adjustment, which differs from historical studies.^35^ The mortality data in this study of patients with AF are consistent with the overall and unselected UK population figures, where the median age at death in 2018–20 was 85.8 years for women and 82.3 years for men, and life expectancy at age 65 years was 21.0 years for women and 18.5 years for men.^36^ This study used a population-based design within primary care to avoid patient selection biases common to registry and hospital-based studies. We restricted the population to address the clinical question of whether gender was useful in risk stratification in AF, considering patients not currently anticoagulated and without an established indication for anticoagulation irrespective of gender. It should be noted that by excluding patients with prior stroke and age ≥75 years, this study is not assessing the full CHA_2_DS_2_-VASc and CHA_2_DS_2_-VA scores, but where gender is clinically relevant to making a decision on oral anticoagulation. Hence, the overall values of performance will not be comparable to studies with unselected inclusion. Restricting the sample also limited complex confounding from various factors in those with high risk, but we cannot exclude impact from unmeasured or unknown confounders. There are also factors that this study did not include that are associated with AF and thromboembolism and may vary according to gender, such as kidney function and body mass index. Biases in outcomes can arise due to delays between disease onset and diagnosis,^37^ so participants in this study were only eligible after an AF diagnosis was clinically made. However, gender disparities are known in the presentation and diagnosis of AF.^24^

Our data confirm that age is the key driver of thromboembolic risk in patients with AF and augments the impact of other comorbidities. Age alone (at a cut-off point of 65 years) was inferior to CHA_2_DS_2_-VA when used as a continuous score, but had numerically similar precision when used as a categorical score. This reinforces that thromboembolic risk is a continuum, and that while risk score categories can guide the prescription of oral anticoagulation, they should not be the absolute determinant. Further, the artificial categorisation of age has the potential to obscure appropriate decision-making for individual patients.^38^ Although risk scoring without the gender criterion had better statistical performance, there were only small differences, which may not impact clinical significance. Detailed assessment of different risk scores was not within scope of this study, which was focused on the value of gender within clinical decision making. It could be argued that the primary outcome for this study should have been the anticoagulant-censored analysis; however, that was prespecified as a sensitivity analysis and was no different to the main analysis for the primary outcome. This study did not collect information on anticoagulation dosage or time in therapeutic range. The presentation, morbidity and management of AF are known to vary across different ethnicity groups.^39,40^ Information on ethnicity was available for 39 619 patients (50.2% of this cohort), of which 1446 (3.7%) were non-white; hence, these data cannot be generalized beyond those of European ancestry.

## Conclusion

Women and men with AF have similar rates of thromboembolic events, such as stroke, arterial or venous clots and vascular dementia after accounting for confounding factors. The rate of the primary composite outcome of all-cause death, ischaemic stroke, or arterial thromboembolism was significantly lower in women than men without prior stroke and aged <75 years, even after censoring for oral anticoagulant use, driven by lower mortality. Clinical risk scores only have a modest ability to predict events in AF, but excluding gender leads to better precision without affecting reclassification or discrimination.

## Supplementary data

Supplementary data are available at *European Heart Journal* online.

## Supplementary Material

ehae539_Supplementary_Data

## Declarations

### Data Availability

No additional data are available at this time. Summary data are available under reasonable request by contacting the corresponding author. The sharing of individual patient data from this study is not possible and would require further ethical approval.
