# Supplementary material for: Gender and contemporary risk of adverse events in atrial fibrillation
Source: Eur Heart J. 2024 Sep 1;45(36):3707–17. doi: 10.1093/eurheartj/ehae539 (PMC11439109; doi:10.1093/eurheartj/ehae539)
Supplement: ehae539_Supplementary_Data [file ehae539_supplementary_data.zip › IMRD_Gender_Supplement_24jun24.docx]

**Impact of gender on the contemporary risk of adverse events in patients with atrial fibrillation**

**Online supplement**

Comparison of risk models 2

Online Figure S1: Study flowchart 3

Online Figure S2: Primary outcome censored for anticoagulation use 4

Online Figure S3: One-year primary outcomes during three time periods 5

Online Figure S4: Crude and adjusted vascular dementia and mortality by gender 6

Online Figure S5: Comparison of risk scores with categorical output 7

Online Figure S6: Comparison of risk scores with age 65 years 8

Online Figure S7: Primary outcome by other CHA_2_DS_2_-VA risk factors 9

Online Figure S8: Primary outcome by CHA_2_DS_2_-VA score 10

Online Figure S9: Secondary outcome according to risk stratification 11

Online Table S1: Global risk stratification for stroke prevention in atrial fibrillation 12

Online Table S2: CODE-EHR framework domains 13

References 16

# Comparison of risk models

**Methods:** Cox hazards regression models for CHA_2_DS_2_-VA and CHA_2_DS_2_-VASc for the primary outcome of all-cause mortality, ischaemic stroke or arterial thromboembolism were run in a randomly-generated derivation set of half the population, stratified by age and gender. CHA_2_DS_2_-VA and CHA_2_DS_2_-VASc models were then compared only in the remaining validation set of patients, using (1) Harrell’s concordance index (C-statistic; proportion of correctly ranked observations in terms of survival times); and (2) Somers’ delta (D-statistic; model fit and agreement between observed and predicted).^1^ Logistic regression models with robust standard error calculation were run in the randomly-generated validation set to assess goodness of fit statistics for CHA_2_DS_2_-VA and CHA_2_DS_2_-VASc without any multivariable adjustment, comparing the odds of the primary outcome for each score value against the reference of zero points. The Bayesian Information Criterion (BIC) assesses overall model fit, identifying the model more likely to have generated the observed outcomes (more negative BIC = better fit), with a difference of >10 providing ‘very strong’ evidence.^2^ Note that patients with prior stroke or age ≥75 years were excluded to focus on a population where gender was a contributor to decision-making on oral anticoagulation; hence these performance figures do not reflect the standard use of these risk scores.

**Results:** Harrell’s C-statistic was significantly higher for CHA_2_DS_2_-VA in the validation set (39,426 patients) compared to CHA_2_DS_2_-VASc: 0.634 (95% CI 0.628-0.640) versus 0.623 (95% CI 0.617-0.630); p for comparison<0.0001. Somers’ D-statistic was also significantly higher for CHA_2_DS_2_-VA than CHA_2_DS_2_-VASc: 0.274 (95% CI 0.261-0.288) versus 0.252 (95% CI 0.238-0.266); p for comparison<0.0001.

BIC values in the validation set were -378152.71 for CHA_2_DS_2_-VA and -377845.82 for CHA_2_DS_2_-VASc, with a difference of 307.89 providing very strong evidence that CHA_2_DS_2_-VA had better model fitting against observed outcomes than CHA_2_DS_2_-VASc.

# Online Figure S1: Study flowchart


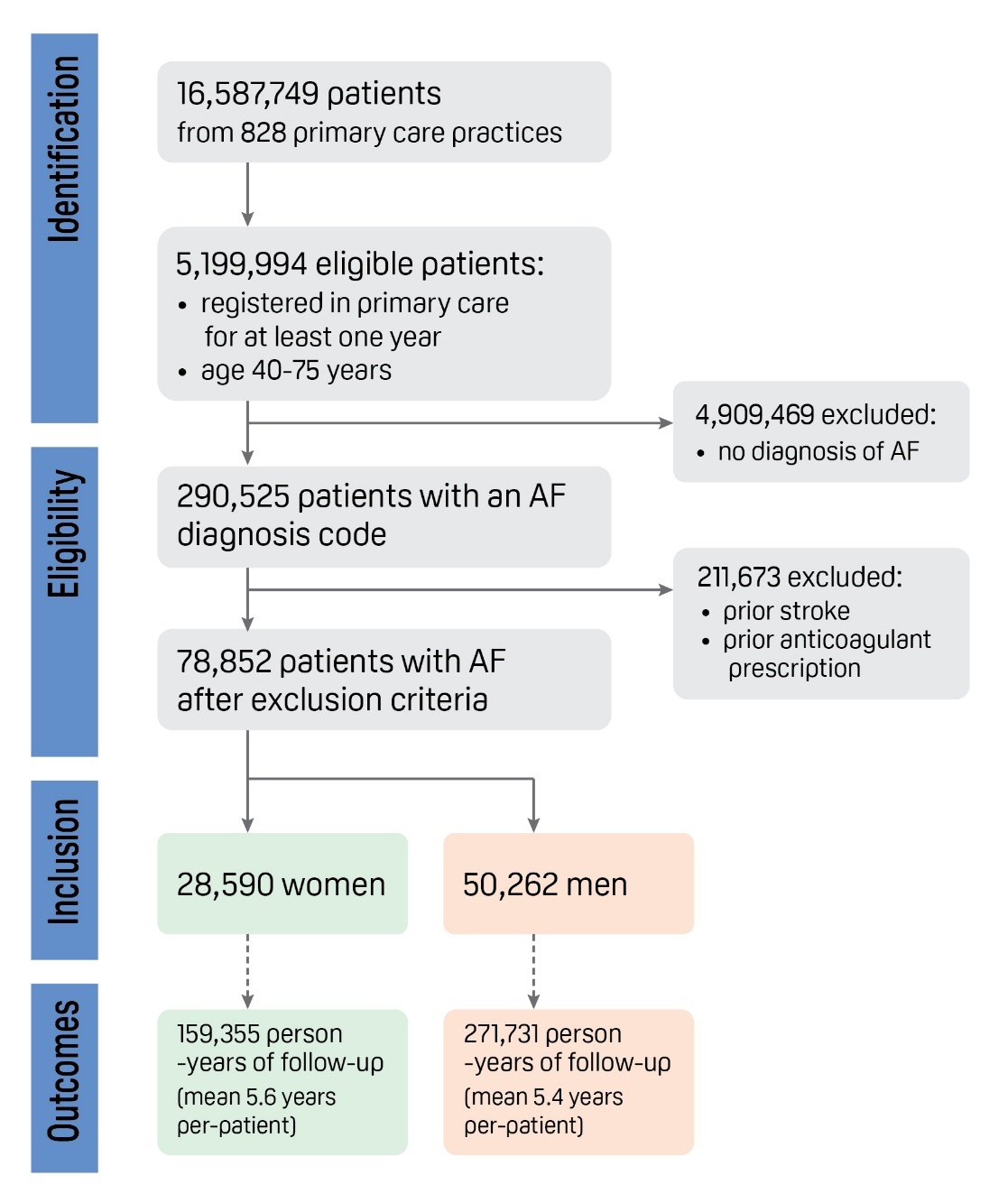


# Online Figure S2: Primary outcome censored for anticoagulation use


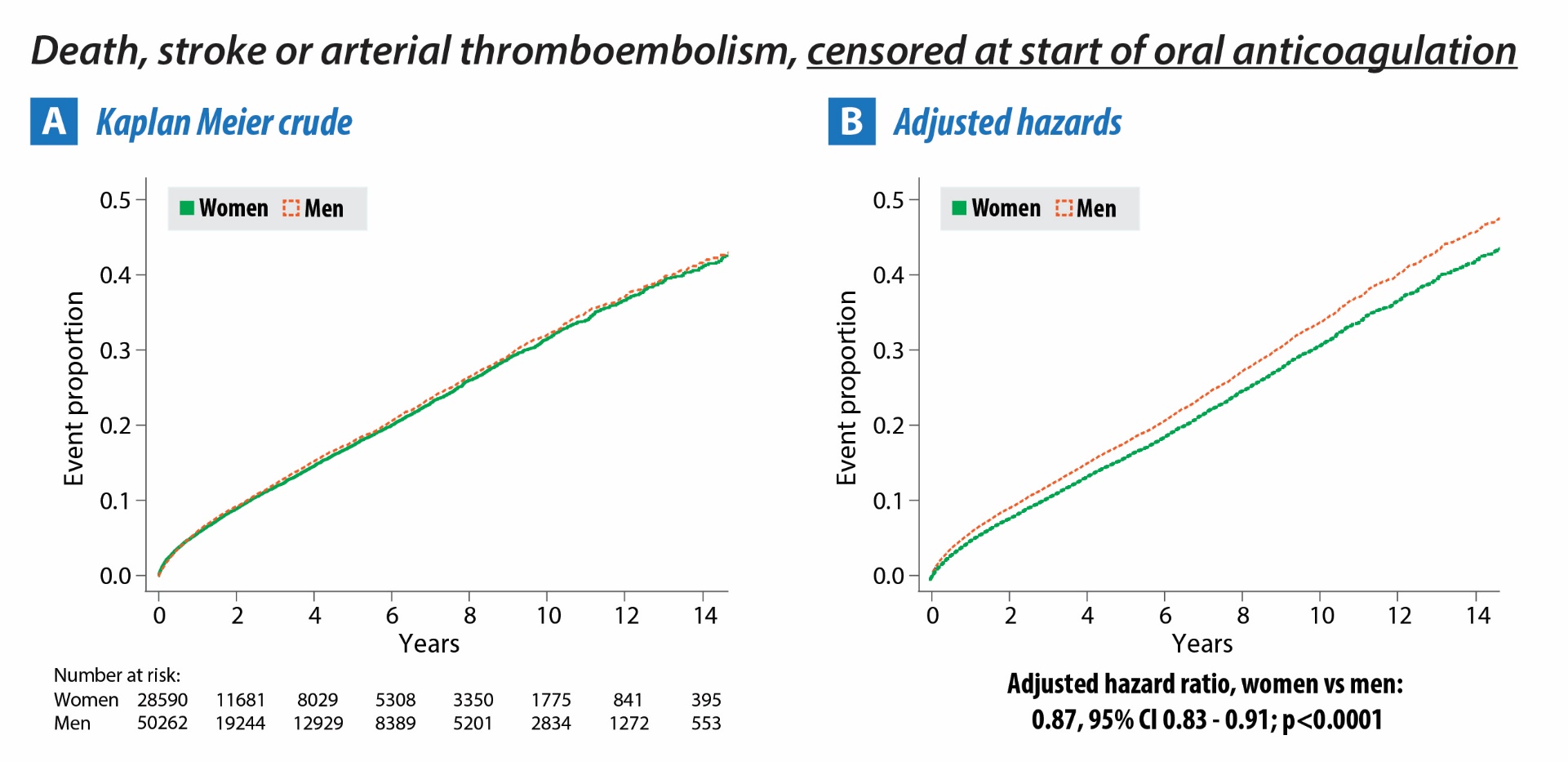


Cumulative event curves for the composite of all-cause mortality, ischaemic stroke or arterial thromboembolism, right-censored at time of commencement of any oral anticoagulant. Presented as crude Kaplan Meier curves (panel A), and after adjustment for age, socioeconomic deprivation status and diagnoses of hypertension, diabetes mellitus, heart failure and vascular disease (panel B) for women (solid green line) and men (dashed orange line). There was no interaction in this model between gender and age as a continuous variable (p-interaction=0.25).

# Online Figure S3: One-year primary outcomes during three time periods


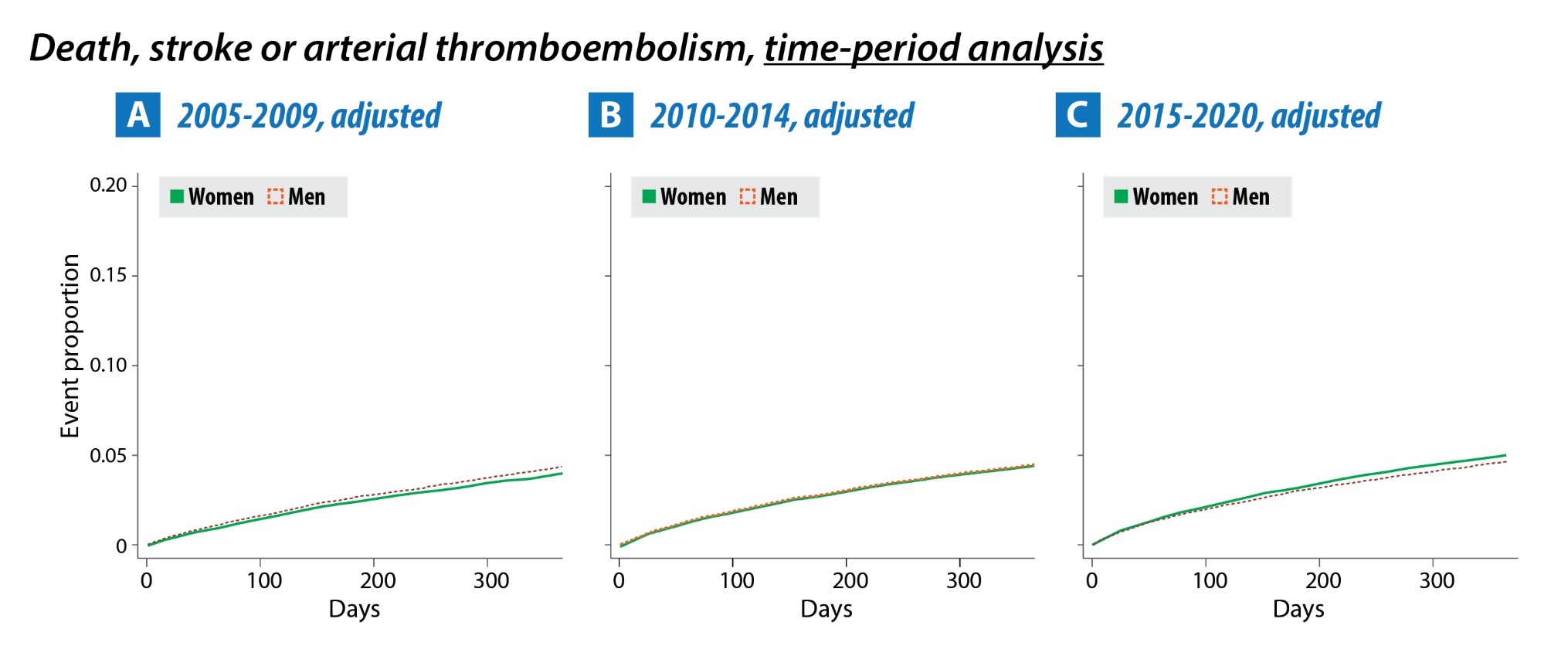


Cumulative event curves for the composite of all-cause mortality, ischaemic stroke or arterial thromboembolism, censored at one year after the date of each patient’s index AF diagnosis and separated into three post-hoc defined time periods. Adjusted for age, socioeconomic deprivation status and diagnoses of hypertension, diabetes mellitus, heart failure and vascular disease for women (solid green line) and men (dashed orange line).

# Online Figure S4: Crude and adjusted vascular dementia and mortality by gender


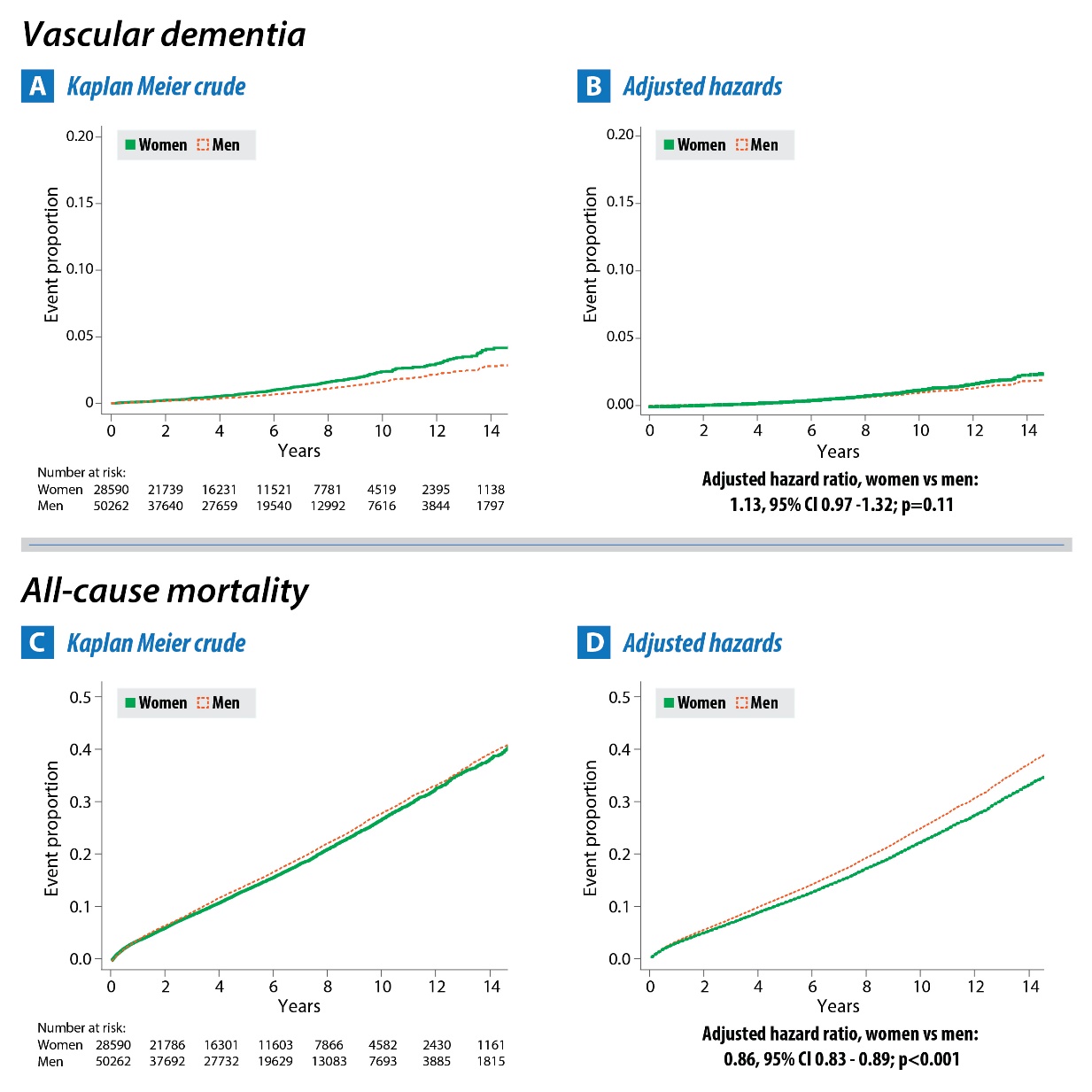


Cumulative event curves for incident vascular dementia and all-cause mortality (note different scales for event proportions). Presented as crude Kaplan Meier curves (panels A and C) and after multivariate adjustment (panels B and D), for women (solid green line) and men (dashed orange line).

# Online Figure S5: Comparison of risk scores with categorical output


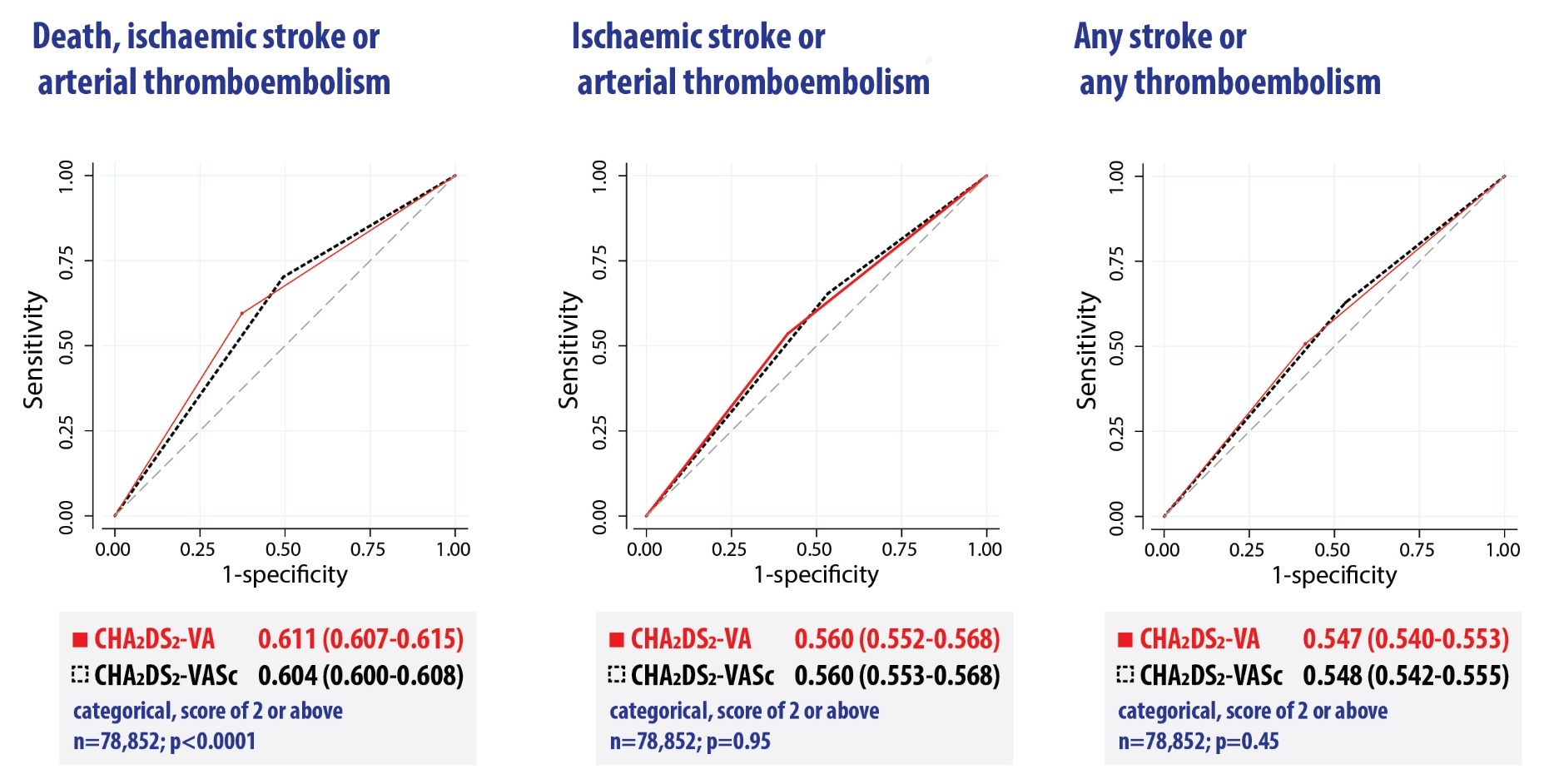


Comparison of the area under the Receiver Operator Characteristic curve for the CHA_2_DS_2_-VA score (solid red line) and CHA_2_DS_2_-VASc score (dashed black line) for each outcome at score cut-off of 2 or above in this population. Higher values indicate better accuracy, with the dashed grey line indicating accuracy no better than chance.

# Online Figure S6: Comparison of risk scores with age 65 years


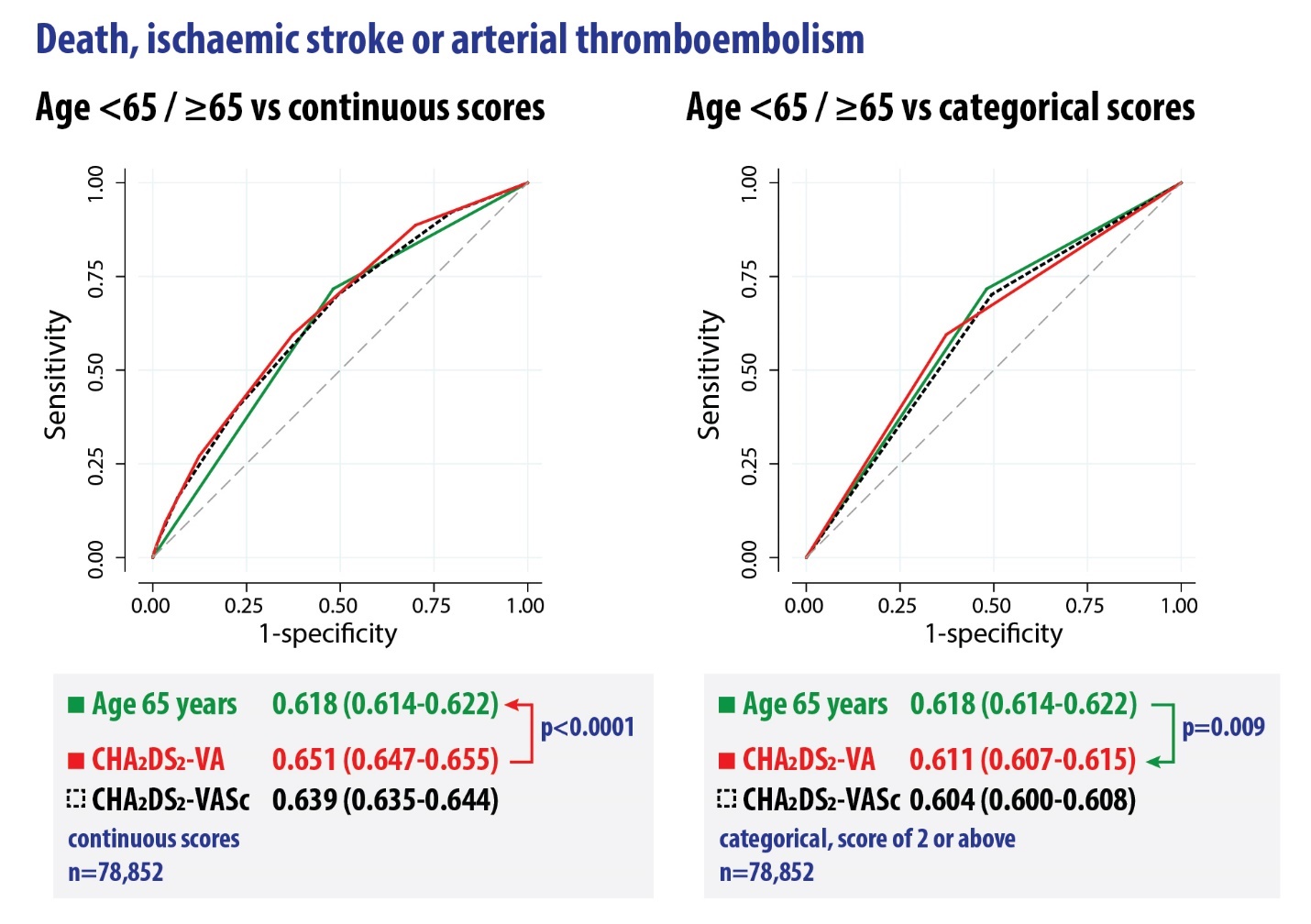


Area under the Receiver Operator Characteristic curve for the primary outcome, comparing CHA_2_DS_2_-VA (solid red line) and CHA_2_DS_2_-VASc (dashed black line) as continuous scores (left panel) and categorical scores (2 or above; right panel) with age at a cut-point of 65 years or older in this population. Higher values indicate better accuracy, with the dashed grey line indicating accuracy no better than chance.

# Online Figure S7: Primary outcome by other CHA_2_DS_2_-VA risk factors


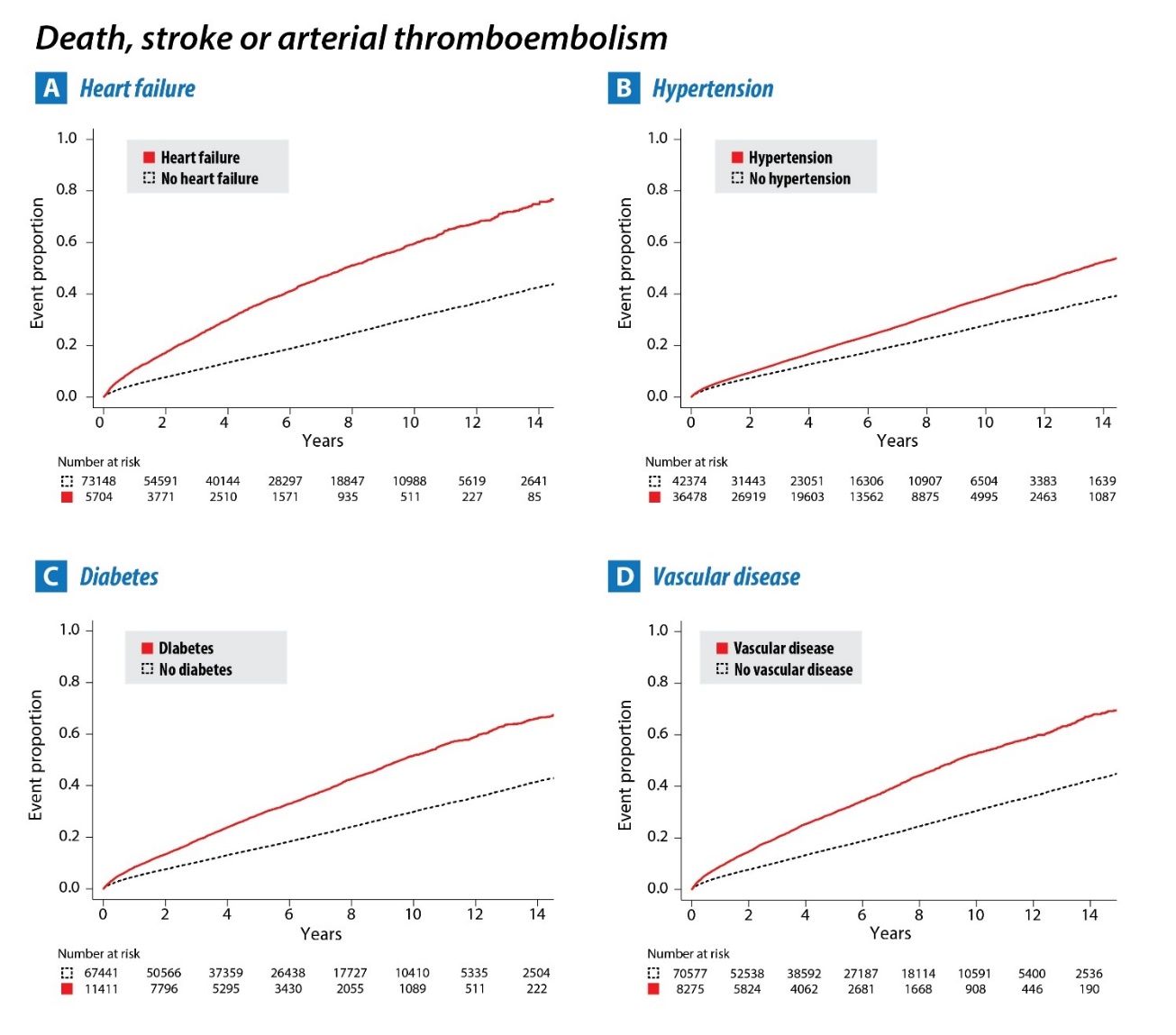


Kaplan Meier event curves for the primary outcome according to other components of the CHA_2_DS_2_-VA score. All comparisons were statistically significant in both crude analysis and after adjustment for gender, age and social deprivation (p<0.01).

# Online Figure S8: Primary outcome by CHA_2_DS_2_-VA score


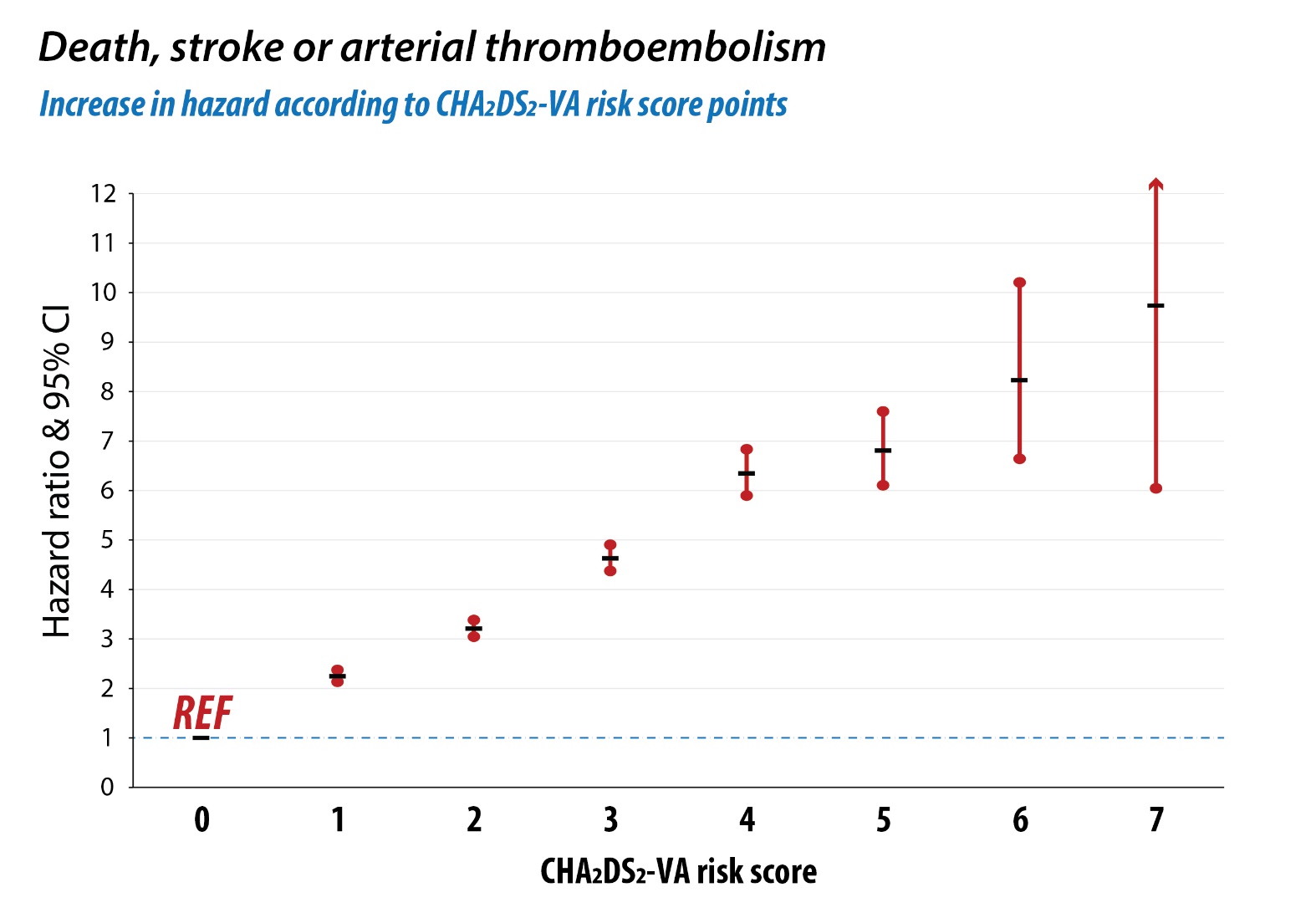


Hazard ratio for the composite of all-cause mortality, ischaemic stroke or arterial thromboembolism for each CHA_2_DS_2_-VA score category in reference to a score of zero (for example, patients with CHA_2_DS_2_-VA score=2 have a 3.2-fold increase in risk compared to CHA_2_DS_2_-VA score=0).

# Online Figure S9: Secondary outcome according to risk stratification


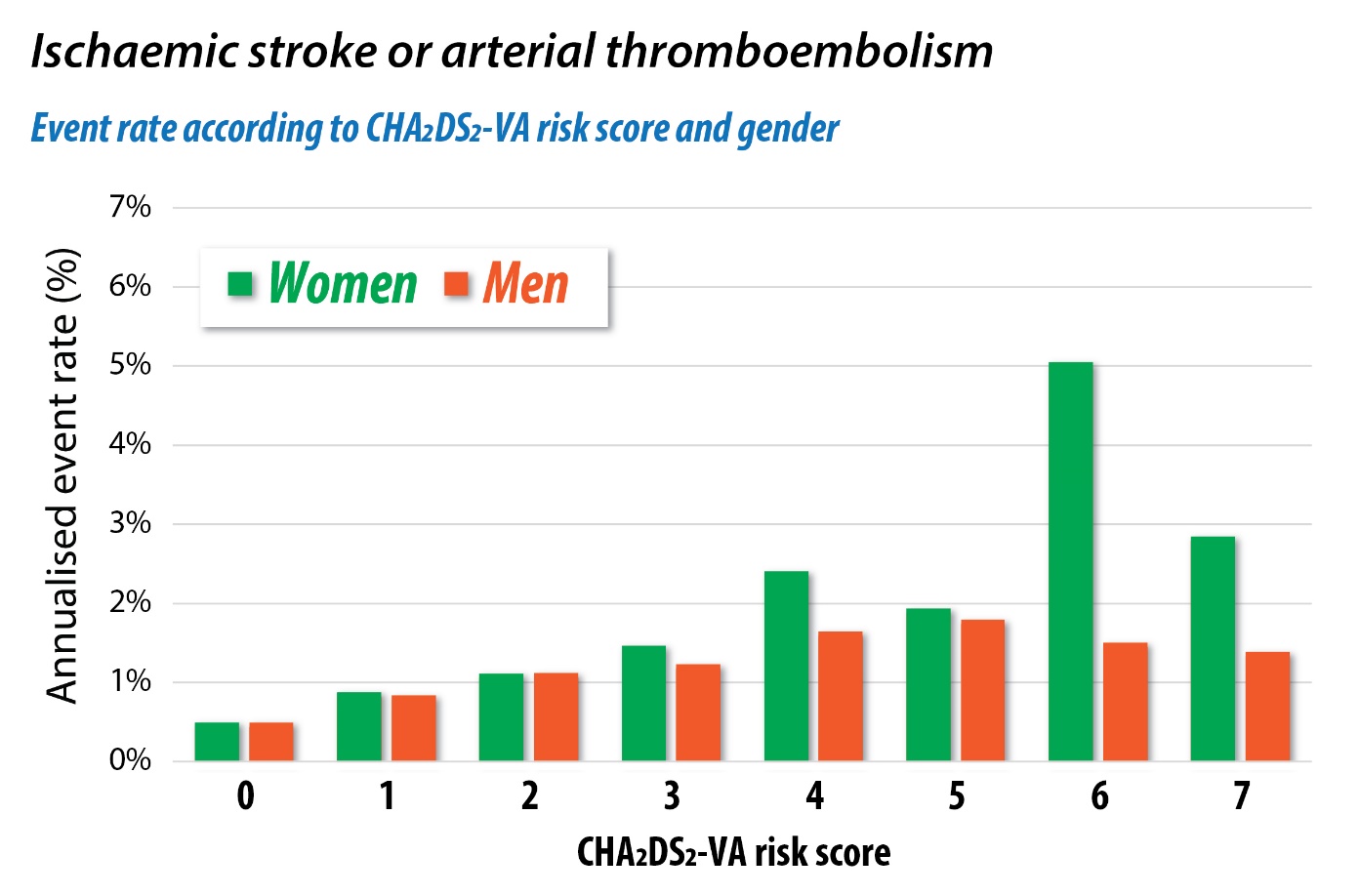


Annualised crude event rate for the composite of ischaemic stroke or arterial thromboembolism for each CHA_2_DS_2_-VA score according to gender.

# Online Table S1: Global risk stratification for stroke prevention in atrial fibrillation

| **Country/Region** | **Relevant atrial fibrillation guideline** | **Score used** | **Class of recommendation for anticoagulation according to score** | **Gender used as a discriminator for anticoagulation prescription** |
| --- | --- | --- | --- | --- |
| Australia/NZ | 2018 National Heart Foundation of Australia and the Cardiac Society of Australia and New Zealand ^3^ | CHA_2_DS_2_-VA | Class I: ≥2 Class IIa: 1 | No |
| Asia-Pacific | 2021 Asia Pacific Heart Rhythm Society ^4^ | CHA₂DS₂-VASc | Anticoagulation indicated for scores ≥2 (men) or ≥3 (women) | No – separate recommendations for women and men |
| Brazil | 2016 Brazilian Society of Cardiology ^5^ | CHA₂DS₂-VASc | Class I: ≥2 for all patients Class IIa: 1 for all patients | Yes |
| Canada | 2020 Canadian Cardiovascular Society and Canadian Heart Rhythm Society ^6^ | CHADS-65 | Class I: ≥65 yrs old  Or <65 with ≥1 CHADS_2_ risk factor | No |
| Europe | 2020 European Society of Cardiology and European Association for Cardio-Thoracic Surgery^7^  *Note upcoming guidelines are due to be published in 2024* | CHA₂DS₂-VASc | Class I: ≥2 (men) or ≥3 (women) Class IIa: 1 (men) or 2 (women) | No – separate recommendations for women and men |
| Japan | 2020 Japanese Circulation Society and Japanese Heart Rhythm Society ^8^ | CHADS_2_ | Class I: ≥2  *CHA_2_DS_2_-VASc and CHA_2_DS_2_-VA are both IIa recommendations* | No |
| USA | 2023 American College of Cardiology, American Heart Association, American College of Chest Physicians and Heart Rhythm Society^9^ | CHA₂DS₂-VASc or other validated scores (i.e. GARFIELD-AF) | Class I: ≥2 (men) or ≥3 (women) Class IIa: 1 (men) or 2 (women) | No – separate recommendations for women and men |

# Online Table S2: CODE-EHR framework domains

| **Domain** | **Category** | **Study information** |
| --- | --- | --- |
| ***Summary*** | ***Additional information is provided for full transparency according to the CODE-EHR best practice framework for structured electronic healthcare record use in clinical research.^10-12^ This study meets all five of the CODE-EHR minimum standards, with three out of five standards meeting preferred criteria (see checklist).*** | |
| **1: Dataset construction and linkage** | Source of dataset | Study data were sourced from IQVIA Medical Research Database (IMRD). IMRD is a UK primary care database containing pseudonymised medical records of patients registered with general practices across the UK using the VISION clinical system. Data extraction and transformation was performed using the Data Extraction for Epidemiological Research (DExtER) tool.^13^ |
|  | Approach to missing data | IMRD uses real world data collected directly from a UK primary care medical records, and therefore missing data is expected. There was no imputation in this study. For deprivation scores, missing data was categorised into a specific category to avoid creating bias in regression models. |
|  | Completeness of follow-up | This study used data from the IMRD database, with outcomes assessed until the earliest of the following time points: (1) Recording of the outcome of interest; (2) Patient censorship due to death or de-registration from their registered practice; (3) Practice censorship due to ceasing of their data contribution to IMRD; and (4) Study end date of 31st December 2020. Censorship happens if a patient de-registers from their General practice during the study, or due to ceasing of their data contribution to IMRD. |
|  | Data linkage | There is no linkage to external data sources in this study. |
| **2: Data fit for purpose** | Origin, process, and purpose of data | IMRD uses real world data collected in primary care practices in the UK. Coded medical data will have been inputted by clinical or administrative staff during routine primary healthcare episodes or after correspondence from secondary care appointments/admissions. High quality data is incentivised in the National Health Service (NHS) through the Quality and Outcomes Framework (QOF), which measures practice performance and is used for billing and reimbursement purposes. |
|  | Coding systems | IMRD uses the Read code coding system. Read Codes are a systematic coding tool that have been used in the NHS since 1985. There were two versions in use during the study period, version 2 (v2) and version 3 (CTV3 or v3). Both versions provide a standard vocabulary for clinicians to record patient findings and management in health and social care systems across primary and secondary care. |
|  | Quality assessment | To ensure data quality in this study, practice data were only included if the practice had contributed to the IMRD database for at least a year and had mortality rates comparable to national averages. A further quality check was conducted in patient inclusion criteria, with patients required to have been registered at their General Practice for at least a year during the time of the study. |
|  | Potential sources of bias | The NHS is a publicly-funded healthcare system and individual clinicians do not receive personal funding for coded healthcare events. The IMRD database encompasses 6% of the population of the UK, with a slight skew towards areas with younger and more affluent patients. Despite this, the prevalence of diseases is consistent with wider UK data, and is generalisable for demographics and major condition prevalence.^14^ At the time of data extraction and analysis there were 832 UK General practices contributing data to IMRD. To ensure data quality, this study only included General Practices one year after they began using the VISION clinical system, or one year after reporting mortality rates comparable to the UK national average. However, this only saw the exclusion of 4 practices out of 832. |
| **3: Disease and outcome definitions** | Definitions | Code lists were developed for inclusion criteria, baseline characteristics and outcome events, and uploaded to a publicly-accessible website (<https://www.birmingham.ac.uk/research/cardiovascular-sciences/research/dare2think/connected-research/connected-research.aspx>). |
|  | Coding manual | The DExtER tool was used to implement the curated code lists for direct data extraction of relevant patients. The coding manual was published online on 23/03/2023. |
|  | Phenotyping approaches | Patients were included in the exposed cohort if there was a code for atrial fibrillation in their record. Exclusion criteria were age 75 years or older on the index date, a history of stroke (any aetiology) in their coded medical record, or a prescription for an oral anticoagulant prior to the index date. |
|  | Validation of coding | The code lists used in this study were developed using the DExtER tool after use and validation in previous studies. All code lists were examined and, where needed, updated prior to analysis. Outcome code lists benefited from a multi-stage validation process undertaken for the DaRe2THINK randomised controlled trial (fully described in the published method paper at <https://doi.org/10.1093/ehjdh/ztac046>).^15^  There is good literature on the use of UK primary care data using validation methods such as case note reporting, direct interrogation of practitioners and against contemporary registries:   - Herrett et al (2009) [https://doi.org/10.1111/Fj.1365-2125.2009.03537.x] conducted a systematic review of the literature to investigate the range of methods used to validate diagnoses in the General Practice Research Database (GPRD); they identified 303 studies that employed data outside of the GPRD for validation and found that the median proportion of cases with a confirmed diagnosis was 89%. - Khan et al (2010) [https://doi.org/10.3399/bjgp10X483562] also reviewed 49 studies to evaluate the validity of diagnoses in the GPRD. The review showed that diagnoses coded in the GPRD electronic record were well recorded when compared against GP questionnaire responses, medical records held at the GP practice, or hospital letters. - Boggon et al (2012) [https://doi.org/10.1002/pds.3374] focused on cancer recording in the GPRD and noted relatively high levels of concordance (>83%) between GP coding and the National Cancer Data Repository (NCDR). |
| **4: Analysis** | Statistical methods | Please see the main paper methods section. |
|  | Machine code | No machine code or algorithms were used in the analysis. |
|  | Internal validation | Cross checks were made of crude unadjusted event rates, incident rates and adjusted data. Regression models were tested for model fit and proportionality, and sensitivity analyses performed as described in the main methods section. |
|  | External validation | The data used in this study was pooled from 828 independent general practices, each with a varying number of General Practitioners (England average in 2022 of 5.5 full-time doctors per practice). No other data sources were used. |
| **5: Ethics and governance** | Consent | IMRD contains fully anonymised data extracted directly from general practice medical records. Data collection for IMRD was approved by the NHS South-East Multicentre Research Ethics Committee. NHS data in England is collected within an ‘opt-out’ approach, meaning that consent is not required. The study was conducted in accordance with the ethical principles set out in the Helsinki Declaration and Recommendations for Good Clinical Practice. |
|  | Data privacy | Data is collected automatically from electronic health records from participating general practices. This includes information about patients’ health such as their diseases, test results and medication, but not their name, address or other information that could directly identify them. Patients who do not wish for their data to be used for research can opt out, through local and national data opt out mechanisms. |
|  | Patient and public involvement | Patients and the public are represented throughout all aspects of ESC Guideline generation, with two patients taking full part as task force members. Patient and public involvement for the health data science aspects is coordinated through the card*AI*c programme at the University of Birmingham/University Hospitals Birmingham NHS Foundation Trust (Clinical & Data Science Forum), and funded by the National Institute for Health & Care Research. |
|  | Data sharing | Summary data are available under reasonable request by contacting the corresponding author. The sharing of individual patient data from this study is not possible and would require further ethical approval. |

# References

1. Newson RB. Comparing the predictive powers of survival models using Harrell’s C or Somers’ D. *Stata J*. 2004;**10**:339-358

2. Raftery AE. Bayesian Model Selection in Social Research. *Sociol Methodol*. 1995;**25**:111-163

3. Brieger D, Amerena J, Attia J, Bajorek B, Chan KH, Connell C, et al. National Heart Foundation of Australia and the Cardiac Society of Australia and New Zealand: Australian Clinical Guidelines for the Diagnosis and Management of Atrial Fibrillation 2018. *Heart Lung Circ*. 2018;**27**:1209-1266

4. Chao T-F, Joung B, Takahashi Y, Lim TW, Choi E-K, Chan Y-H, et al. 2021 Focused update of the 2017 consensus guidelines of the Asia Pacific Heart Rhythm Society (APHRS) on stroke prevention in atrial fibrillation. *J Arrhythmia*. 2021;**37**:1389-1426

5. Magalhães LP, Figueiredo MJO, Cintra FD, Saad EB, Kuniyoshi RR, Menezes Lorga Filho A, et al. Executive Summary of the II Brazilian Guidelines for Atrial Fibrillation. *Arq Bras Cardiol*. 2016;**107**:501-508

6. Andrade JG, Aguilar M, Atzema C, Bell A, Cairns JA, Cheung CC, et al. The 2020 Canadian Cardiovascular Society/Canadian Heart Rhythm Society Comprehensive Guidelines for the Management of Atrial Fibrillation. *Can J Cardiol*. 2020;**36**:1847-1948

7. Hindricks G, Potpara T, Dagres N, Arbelo E, Bax JJ, Blomström-Lundqvist C, et al. 2020 ESC Guidelines for the diagnosis and management of atrial fibrillation developed in collaboration with the European Association for Cardio-Thoracic Surgery (EACTS): The Task Force for the diagnosis and management of atrial fibrillation of the European Society of Cardiology (ESC) Developed with the special contribution of the European Heart Rhythm Association (EHRA) of the ESC. *Eur Heart J*. 2021;**42**:373-498

8. Ono K, Iwasaki Y-k, Akao M, Ikeda T, Ishii K, Inden Y, et al. JCS/JHRS 2020 Guideline on Pharmacotherapy of Cardiac Arrhythmias. *Circ J*. 2022;**86**:1790-1924

9. Joglar JA, Chung MK, Armbruster AL, Benjamin EJ, Chyou JY, Cronin EM, et al. 2023 ACC/AHA/ACCP/HRS Guideline for the Diagnosis and Management of Atrial Fibrillation: A Report of the American College of Cardiology/American Heart Association Joint Committee on Clinical Practice Guidelines. *Circulation*. 2024;**149**:e1-e156

10. Kotecha D, Asselbergs FW, Achenbach S, Anker SD, Atar D, Baigent C, et al. CODE-EHR best practice framework for the use of structured electronic healthcare records in clinical research. *BMJ*. 2022;**378**:e069048

11. Kotecha D, Asselbergs FW, Achenbach S, Anker SD, Atar D, Baigent C, et al. CODE-EHR best-practice framework for the use of structured electronic health-care records in clinical research. *Lancet Digit Health*. 2022;**4**:e757-e764

12. Kotecha D, Asselbergs FW, Achenbach S, Anker SD, Atar D, Baigent C, et al. CODE-EHR best practice framework for the use of structured electronic healthcare records in clinical research. *Eur Heart J*. 2022;**43**:3578-3588

13. Gokhale KM, Chandan JS, Toulis K, Gkoutos G, Tino P, Nirantharakumar K. Data extraction for epidemiological research (DExtER): a novel tool for automated clinical epidemiology studies. *Eur J Epidemiol*. 2021;**36**:165-178

14. Candore G, Hedenmalm K, Slattery J, Cave A, Kurz X, Arlett P. Can We Rely on Results From IQVIA Medical Research Data UK Converted to the Observational Medical Outcome Partnership Common Data Model?: A Validation Study Based on Prescribing Codeine in Children. *Clin Pharmacol Ther*. 2020;**107**:915-925

15. Wang X, Mobley AR, Tica O, Okoth K, Ghosh RE, Myles P, et al. Systematic approach to outcome assessment from coded electronic healthcare records in the DaRe2THINK NHS-embedded randomized trial. *Eur Heart J - Dig Health*. 2022;**3**:426-436
